# Mobile Phones and Multiple Sclerosis – A Nationwide Cohort Study in Denmark

**DOI:** 10.1371/journal.pone.0034453

**Published:** 2012-04-30

**Authors:** Aslak Harbo Poulsen, Egon Stenager, Christoffer Johansen, Joan Bentzen, Søren Friis, Joachim Schüz

**Affiliations:** 1 Institute of Cancer Epidemiology, Danish Cancer Society, Copenhagen, Denmark; 2 The Danish MS Registry, Copenhagen University Hospital, Copenhagen, Denmark; 3 National Institute of Public Health, University of Southern Denmark, Copenhagen, Denmark; 4 International Agency for Research on Cancer (IARC), Section of Environment and Radiation, Lyon, France; 5 Institute of Regional Health Services, University of Southern Denmark, Odense, Denmark; 6 MS Clinic of Southern Jutland (Sønderborg, Vejle, Esbjerg), Sønderborg Hospital, Sønderborg, Denmark; Kyushu University, Japan

## Abstract

We investigated the risk of, prognosis of and symptoms of multiple sclerosis (MS) among all Danish residents who owned a mobile phone subscription before 1996. Using the Danish Multiple Sclerosis Registry and Civil Registration System, study subjects were followed up for MS through 2004. Poisson models were used to calculate incidence rate ratios (IRR, age range: 18–64 years) and mortality rate ratios (MRR, age range: 18+) and to compare presenting symptoms among subscribers and all non-subscribers. A total of 405 971 subscription holders accrued four million years of follow up, with men accounting for 86% of the observation time. Among subscription holding men, the IRR of MS was close to unity, overall as well as 13+ years after first subscription (IRR 1.02, 95% CI: 0.48–2.16). Among women, the IRR was 3.43 (95% CI: 0.86–13.72) 13+ years after first subscription, however, based on only two cases. Presenting symptoms of MS differed between subscribers and non-subscribers (p = 0.03), with slightly increased risk of diplopia in both genders (IRR: 1.38, 95% CI: 1.02–1.86), an increased risk of fatigue among women (IRR: 3.02, 95% CI: 1.45–6.28), and of optic neuritis among men (IRR: 1.38, 95% CI: 1.03–1.86). Overall the MRR was close to one (MRR: 0.91, 95%CI 0.70–1.19) among MS-patients with a subscription and although we observed some increased MRR estimates among women, these were based on small numbers. In conclusion, we found little evidence for a pronounced association between mobile phone use and risk of MS or mortality rate among MS patients. Symptoms of MS differed between subscribers and nonsubscribers for symptoms previously suggested to be associated with mobile phone use. This deserves further attention, as does the increased long-term risk of MS among female subscribers, although small numbers and lack of consistency between genders prevent causal interpretation.

## Introduction

Usage of mobile phones has changed dramatically in the last two decades, with steeply increasing number of users and decreasing age at first use. The radiofrequency electromagnetic fields (RF-EMF) emitted from mobile phones held to the head penetrates up to six cm into the brain [1], [2] and there remains concern about potential adverse health effects. Epidemiological studies have largely been on a potential risk of neoplasms, particularly of the brain, and a working group at the International Agency for Research on Cancer (IARC) has recently classified RF-EMF as possibly carcinogenic [3]. For other diseases of the central nervous system (CNS) and head, very few results are available. One of the most frequent CNS diseases is multiple sclerosis (MS), characterized by progressive demyelination of the axons of the CNS causing a wide range of neurological symptoms. MS is typically diagnosed in persons in their thirties. Only symptomatic treatment is available, but survival is good [4], making MS a leading cause of disability in younger adults in the developed world [5]. In Denmark, the prevalence of MS in 2005 was 154.5 per 100 000 [6]. There are few established risk factors for MS, but leakage across the blood brain barrier is prominent in the pathophysiology of the disease. Therefore, it is of interest that a Swedish research group has observed leakage of the blood brain barrier in rats exposed to 900 MHz field from a GSM phone [7]–[10]. Attempts to replicate these findings have, however, failed and the overall evidence remains controversial [11]–[14]. Notwithstanding this, the results may have caused concern in MS patients as to whether mobile phones have an impact on their disease. A recent nationwide Danish cohort study examined the association between mobile phone use and risk of various CNS diseases based on information on mobile phone subscription status prior to 1996 [15]. The study found no increased risk of MS overall or in mobile phone subscribers with 10 or more years of subscription. Information on MS was, however, obtained from the Danish National Patient Register [16], which only provides few details on each specific case and thus is prone to disease misclassification [17] and underreporting, especially on date of diagnosis. The present population-based epidemiologic study used the same cohort but with information on MS from the Danish Multiple Sclerosis Registry [18], which provides more precise case identification and allows analysis of the influence of exposure status on the symptoms and course of the disease. To our knowledge, this is the first study examining the long term effects of mobile phone use on risk and prognosis of MS.

## Methods

### Ethics Statement

The study was approved by the Danish ethical committee system (KF 01–075/96), the Danish Data Protection Board (1996–1200–121, 2009–41–3886), and the Danish Ministry of Justice (Jnr. 1996–760–0219). In accordance with Danish law informed consent was not obtained as the study was entirely register-based and did not involve biological samples from, or contact with study participants.

### Study Population

We conducted this study within the population of Denmark during the period 1987–2004. All Danish citizens are assigned a unique personal identification number at birth by the Danish Central Population Registry (CPR), which keeps complete information about the date of birth, gender, vital status, migration and current and former addresses of all Danes [19]. This identification number is applied universally in all contacts within the health care systems in Denmark allowing individuals to be tracked over time in and across all Danish administrative and health registers.

### Ascertainment of MS Cases

MS cases were identified from the Danish Multiple Sclerosis Registry [18], [20] established in 1956. The register contains information on more than 90% of all MS patients in Denmark since 1949 and is considered to have a validity of 94%. For each patient the medical records have been evaluated and the year of the first symptom has been assessed. Furthermore, the first symptom(s) for each patient in the register are recorded, and multiple simultaneous symptoms are allowed. For the present paper, only definite and probable diagnoses according to the Poser criteria [21] were included.

### Exposure Assessment

Records of all (723 421) mobile phone subscriptions in Denmark during the period 1982 (when this service was established) until the end of 1995 were obtained from the Danish network operatoros. Details of the cleaning process of these data has been reported previously [15], [22]–[24]: Briefly, 200 507 corporate subscriptions were deleted and 102 828 records were deleted for other reasons including errors in matching variables and duplicate records (persons with multiple subscriptions) leaving a cohort of 420 086 private mobile phone subscription holders. Since handheld mobile phones first became available in Denmark in 1987, all subscription periods were left truncated to 1 January 1987, further deleting four persons from the dataset. The unexposed population was obtained by subtracting the number of MS-cases and exposed persons from the Danish population count by age and gender for each year of the study. Only persons, who turned 18 years before January 1^st^ 1996, i.e., end of exposure period, were included in the analysis.

### Risk of MS Among Subscription Holders

In analyses of risk of MS and presenting symptoms among mobile phone subscription holders, symptom free subjects entered the study population on 1 January 1987 or age 18 years, whichever occurred latest. Follow-up ended at date of MS diagnosis, age 65 years, death, emigration from Denmark or 31 December 2004, whichever came first. Exposed person time was further categorized based on duration of follow-up since date of first subscription (<1, 1–3, 4–6, 7–9, 10–12, 13+ years). In a subanalysis, we used July 1^st^ in the year of the first recorded symptom(s) as endpoint. Due to the retrospective nature of debut data follow-up was, for this analysis, terminated on 31 December 2000 allowing four years until 2004 to identify patients with first symptoms in 2000 or earlier. In these analyses, we excluded 13 573 subscription holders who obtained their subscription after age 65 years, 30 with MS symptoms before age 18 years, 366 with symptoms before 1987 and 142 diagnosed with MS before getting a subscription, yielding a population of 405 971 mobile phone subscription holders.

### Risk of Death Among MS-patients

In analyses of risk of death among MS patients using mobile phone, MS patients diagnosed between age 18 and 65 years in 1980 or later entered the study population at date of diagnosis or 1 January 1987, whichever came latest. Follow-up ended at date of death, emigration from Denmark or on 31 December 2004, whichever came first. A total of 7420 MS-patients met the inclusion criteria, of whom 717 were subscription holders. For these patients, exposed person-years were cumulated from date of diagnosis or date of subscribing, whichever came last, and subdivided into five categories (<1, 1–3, 4–6, 7–9, 10+ years).

### Likelihood of Subscribing for a Mobile Phone Among MS Patients

To evaluate a potentially reverse association, we analysed MS as an explanatory factor for obtaining a subscription in the 5 050 MS patients with a first symptom between age 18 and 65 years in the period 1980 to 1995. Entry and exit criteria were as for the main analysis, except that subjects were censored at the date of subscription acquisition, or on 31 December 1995, and not at the time of the MS-diagnosis. Time after first MS-symptom was subdivided according to diagnostic status (1st symptom, diagnosis), and years since first symptom and since diagnosis (<1, 1–3, 4–6, 7–9, 10+ years).

### Statistical Analysis

Log-linear Poisson regression analysis was used to compute incidence and mortality rate ratios (IRRs or MRRs) for MS diagnosis, MS debut and risk of death in MS patients among mobile phone subscription holders compared to non-subscribers. The analyses were adjusted for gender, age (in incidence analyses: 18–29, 30–39, 40–49 and 50–65 years; in mortality analyses:18–29, 30–39, 40–49, 50–59, 60–69, 70–79 and 80+ years) and individual calendar year (1987 to 2004 by increments of 1 year). Subjects were allowed to change between categories of covariates and exposure variables over time. When analysing risk of death, years since diagnosis was included as a linear covariate.

The presenting symptoms of MS among subscribers and nonsubscribers were compared by analysing MS diagnoses with different initial symptoms as competing risks in a Poisson model as above with independent gender, age and period dependency for each symptom.

For date variables with missing day values, the 15th of the respective month was used and when the month was missing, July 1st was used. As only the year of first symptom was available, the date of first symptom was set to July 1st of the given year or the actual date of diagnosis, whichever came first. The statistical analyses were performed in SAS 9.1.

## Results

**Figure 1:**
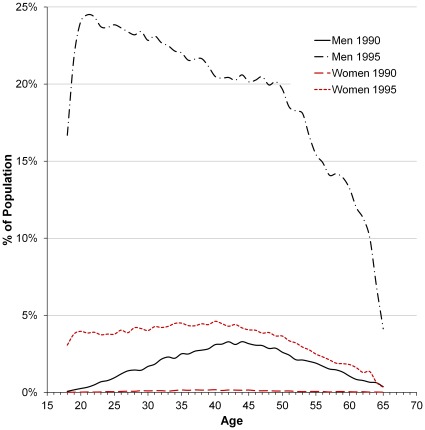
Mobile phone subscription penetration in the Danish population by age in 1990 and 1995.

The gender and age profile of new subscription holders changed dramatically over the course of the exposure (mobile phone subscription) period (Fig. 1). In 1990, 0.1% of all women and 1.9% of all men aged 18–65 years owned a subscription in their own name, with the highest proportion in middle-aged men. In 1995, 3.6% of all women had a subscription with penetration decreasing gradually above age 45 years. In men the penetration was above 20% from age 19 to 47 years, decreasing rapidly in the older ages.

### Risk of MS Among Subscription Holders

The 405 971 subscription holders accrued 4 063 040 years of follow up (mean 10.0 years; maximum 18.0 years). The overall risks of MS in subscription holding women and men were 1.02 (95% CI: 0.83–1.24) and 1.11 (95% CI: 0.98–1.26), respectively (Table 1). Stratification of subscription holders by years since first subscription showed a slight overall increase in IRR of MS among male subscribers of 10 to 20% in the period from 1–9 years after first subscription, however, there was no elevation in risk before or after that interval. Among women, elevated IRRs were seen in the first year after subscription (1.61; (95% CI: 0.93–2.79, n = 13) and beyond 10 years of subscription (2.08; 95%CI: 1.08–4.01, n = 9), whereas the risk estimates were close to one in the intervening period. Further restricting the follow-up period to 13+ years showed an even higher estimate (IRR: 3.43, 95%CI: 0.86–13.72), however based on only two cases. In analysis of both genders, IRRs ranged from 1.04 to 1.09, except in the stratum of 13+ years of subscribing where there was a 26% increased risk of MS (95% CI 0.65–2.43).

**Table 1:** Adjusted rate ratios for Multiple Sclerosis among private subscribers to mobile phones in Denmark, 1987–1995, followed up through 2004.

		Women			Men			Total		
		Personyears	MS	IRR 95% CI	Personyears	MS	IRR 95% CI	Personyears	MS	IRR 95% CI
**Until diagnosis**										
**Non subscribers**		27 517 890	3 531	Reference	25 309 803	1 517	Reference	52 827 693	5 048	Reference
**Subscription holders**		572 177	102	1.02 (0.83–1.24)	3.490.862	304	1.11 (0.98–1.26)	4 063 040	406	1.06 (0.96–1.18)
**Years of subscription**										
<1	60 044		13	1.61 (0.93–2.79)	344 413	18	0.91 (0.57–1.46)	404 457	31	1.09 (0.76–1.56)
1–3	176 918		26	1.00 (0.68–1.48)	1 012 944	70	1.12 (0.87–1.44)	1 189 862	96	1.05 (0.85–1.29)
4–6	171 243		28	0.84 (0.58–1.22)	975 610	100	1.21 (0.98–1.50)	1 146 853	128	1.08 (0.90–1.29)
7–9	139 559		26	0.91 (0.61–1.34)	840 374	91	1.11 (0.89–1.39)	979 932	117	1.04 (0.86–1.26)
10–12	20 847		7	1.87 (0.89–3.94)	234 295	18	0.88 (0.55–1.41)	255 142	25	1.04 (0.70–1.54)
13+	3 567		2	3.43 (0.86–13.72)	83 227	7	1.02 (0.48–2.16)	86 794	9	1.26 (0.65–2.43)
10+	24 414		9	2.08 (1.08–4.01)	317 522	25	0.92 (0.61–1.37)	341 936	34	1.09 (0.77–1.53)
**Until 1st symptom**										
**Non subscribers**		21 929 622	3 336	Reference	20 599 730	1 485	Reference	42.529.353	4 821	Reference
**Subscription holders**		351 972	57	0.94 (0.72–1.23)	2 261 285	195	1.10 (0.94–1.28)	2 613 258	252	1.05 (0.92–1.19)
**Years of subscription**										
<1		60 012	7	0.70 (0.33–1.48)	344 332	33	1.33 (0.94–1.90)	404 344	40	1.11 (0.81–1.52)
1–3		176 836	30	0.98 (0.69–1.41)	1 012 638	84	1.06 (0.84–1.33)	1 189 474	114	1.02 (0.84–1.24)
4–6		104 217	17	0.93 (0.58–1.51)	705 980	65	1.09 (0.84–1.41)	810 197	82	1.06 (0.84–1.32)
7–9		8 703	1	0.73 (0.10–5.16)	138 245	11	1.06 (0.59–1.93)	146 948	12	1.05 (0.60–1.86)
10+		2 204	2	6.75 (1.69–27.05)	60 091	2	0.50 (0.13–2.02)	62 295	4	0.99 (0.37–2.65)

Analysing until year of first symptom yielded similar results when comparing ever versus never subscribing. However, among men we observed a 33% increased risk of having the first symptom within one year of obtaining a subscription. In subsequent years there was little deviation from unity, except in the strata of 10+ years of usage where the risk was reduced by half but based on only two cases (IRR: 0.50, 95%CI 0.13–2.02). Among women, there was a 30% decreased risk in the first year after subscribing and the only estimate above one was for 10+ years of subscribing where there was a more than six fold increased risk, however based on only two cases.

### Presenting Symptoms

Presenting symptoms of MS differed significantly between subscribers and non-subscribers (Table 2) overall and among women, but not among men (respective p-values: 0.03, 0.02 and 0.46). When examining the individual symptoms, there was a slightly increased risk of diplopia (double vision) in both genders; with an IRR of 1.38 (95% CI: 1.02-1.86, n = 54) in combined analysis of men and women. Among women, there was also an increased risk of fatigue (IRR: 3.02, 95% CI: 1.45–6.28, n = 8), and among men there was increased risk of optic neuritis (IRR: 1.38, 95% CI: 1.03–1.86, n = 58).

**Table 2:** Adjusted rate ratios for Multiple Sclerosis with different first symptoms among private subscribers to mobile phones in Denmark, 1987–1995, followed up through 2004.

	Women			Men			Total		
	Unexposedcases	Exposedcases	IRR 95% CI	Unexposedcases	Exposedcases	IRR 95% CI	Unexposedcases	Exposedcases	IRR 95% CI
Person years	27 517 890	572 177		25 309 803	3 490 862		52 827 693	4 063 040	
Cerebellar symptoms	326	4	0.40 (0.15–1.08)	138	21	0.81 (0.51–1.30)	464	25	0.67 (0.44–1.01)
Diplopia	300	14	1.59 (0.92–2.72)	157	40	1.32 (0.92–1.89)	457	54	1.38 (1.02–1.86)
Optic neuritis	694	23	1.08 (0.71–1.64)	217	58	1.38 (1.03–1.86)	911	81	1.24 (0.97–1.57)
Pyramidal dysfunction	837	17	0.71 (0.44–1.15)	452	80	1.00 (0.78–1.27)	1 289	97	0.90 (0.73–1.12)
Sensory symptoms	1 836	50	0.92 (0.69–1.22)	653	130	1.02 (0.84–1.24)	2 489	180	0.98 (0.84–1.15)
Sphincter control	116	1	0.27 (0.04–1.97)	63	15	1.20 (0.68–2.14)	179	16	0.95 (0.56–1.61)
Vertigo	226	8	0.95 (0.47–1.92)	61	12	0.79 (0.43–1.47)	287	20	0.85 (0.53–1.35)
Fatigue	74	8	3.02 (1.45–6.28)	40	11	1.09 (0.56–2.12)	114	19	1.61 (0.96–2.69)
Other/Unstated symptoms	355	8	1.28 (0.63–2.59)	145	23	1.30 (0.82–2.06)	500	31	1.30 (0.89–1.89)

### Risk of Death Among MS-patients

The 717 subscription holding MS-patients accrued 4 934 years of exposed follow-up from 1987 onwards (mean: 6.9 years; maximum 18.0 years). The overall MRRs of death were 1.27 (95% CI: 0.77–2.09) and 0.79 (95% CI: 0.58–1.09) among female and male MS-patients, respectively, with a subscription (Table 3) compared to patients without subscription. In combined analysis of the two genders, the IRR was 0.91 (95% CI: 0.70–1.19). Among men, the risk of death was reduced by half or more in the first four years of exposed follow-up time, especially in the interval from 1–3 years after which the death risk estimates approached unity. Among women, the death estimates varied more, but did not deviate significantly from unity, except among female MS patients with 7–9 years of mobile phone usage after diagnosis (MRR, 2.44; 95% CI: 1.20–4.98; n = 8). The combined analysis of the two genders revealed a 40% decreased MRR in the first four years and a 41% increase seven to nine years after subscribing.

**Table 3:** Risk of death among Multiple Sclerosis patients with private subscriptions to mobile phones in Denmark. 1987–1995, followed up through 2004.

	Women			Men			Total		
	Person years	Deaths	MRR 95% CI	Person years	Deaths	MRR 95% CI	Person years	Deaths	MRR 95% CI
**Non subscribers**	39 575	400	Reference	18 333	306	Reference	57 908	706	Reference
**Subscription holders**	1 339	16	1.27 (0.77–2.09)	3 595	45	0.79 (0.58–1.09)	4 934	61	0.91 (0.70–1.19)
**Years of subscription after MS-diagnosis**									
<1	190	2	1.35 (0.33–5.45)	509	2	0.40 (0.10–1.64)	700	4	0.59 (0.22–1.59)
1–3	487	4	1.06 (0.39–2.85)	1 305	7	0.47 (0.22–1.00)	1 792	11	0.58 (0.32–1.06)
4–6	382	2	0.57 (0.14–2.00)	934	14	0.91 (0.53–1.57)	1 317	16	0.90 (0.54–1.48)
7–9	246	8	2.44 (1.20–4.98)	645	16	1.06 (0.63–1.78)	891	24	1.41 (0.93–2.15)
10+	33	0	–	202	6	0 93 (0.41–2.12)	235	6	0.95 (0.42–2.15)

### Likelihood of Subscribing for a Mobile Phone Among MS Patients

The 5 050 MS patients accrued 29 993 person years from 1987 to 1995 (mean, 5.9 years; maximum 9.0 years). Among women, there was no increased likelihood of obtaining a subscription after first symptoms or in the first year after being diagnosed with MS (Table 4). Beyond that there was a tendency for an increased likelihood of obtaining a subscription (IRRs ranging from 1.18–1.44). Among men, there was no overall association with first symptoms or diagnosis of MS. However, in the first four years after getting the symptom, the IRRs were 25% elevated and beyond that they were around 20% decreased. Once diagnosed with MS, there was no apparent increased likelihood of acquiring a mobile phone subscription, except in the period 1–3 years after diagnosis (IRR: 1.22; 95% CI: 0.97–1.54). In combined analysis of the two genders, there was no indication of an increased likelihood of acquiring a subscription after first symptoms or diagnosis. Except for a 26% increased tendency for subscription 1–3 years after diagnosis (95% CI: 1.04–1.53), there was no apparent trends between first symptoms or MS diagnosis and mobile phone subscription in the combined analysis of the two genders.

**Table 4:** Likelihood of getting subscription after diagnosis or first symptoms of Multiple Sclerosis among private subscribers to mobile phones in Denmark, 1987–1995.

	Women				Men			Total		
	Personyears		Subscrpt	IRR 95% CI	Person years	Subscrpt	IRR 95% CI	Person years	Subscrpt	IRR 95% CI
**Non- MS**	14 376 975		60 019	Reference	14 188 947	338 243	Reference	28 565 922	398 262	Reference
**Diagnostic stage**										
Symptoms	8 604		41	1.00 (0.73–1.35)	3 857	97	1.04 (0.85–1.27)	12 461	138	1.04 (0.88–1.22)
Diagnosis	11 172		86	1.22 (0.99–1.51)	6 360	193	1.00 (0.87–1.15)	17 532	279	1.07 (0.95–1.21)
**Years since 1st MS-symptom (Censor at diagnosis)**										
<1	1 581	7		1.00 (0.48–2.10)	742	21	1.24 (0.81–1.90)	2 323	28	1.17 (0.81–1.69)
1–3	3 168	8		0.61 (0.31–1.23)	1 512	40	1.25 (0.92–1.71)	4 680	48	1.07 (0.80–1.42)
4–6	2 052	12		1.31 (0.74–2.30)	874	17	0.85 (0.53–1.36)	2 926	29	1.00 (0.69–1.43)
7–9	1 190	6		0.96 (0.43–2.14)	501	11	0.78 (0.43–1.40)	1 692	17	0.84 (0.52–1.35)
10+	612	8		1.41 (0.70–2.81)	228	8	0.82 (0.41–1.64)	840	16	1.07 (0.65–1.74)
**Years since MS-diagnosis**										
<1	1 629	6		0.67 (0.30–1.50)	865	24	1.02 (0.69–1.52)	2 494	30	0.93 (0.65–1.33)
1–3	4 068	31		1.31 (0.92–1.87)	2 252	72	1.22 (0.97–1.54)	6 320	103	1.26 (1.04–1.53)
4–6	2 849	23		1.44 (0.96–2.17)	1 735	48	0.96 (0.72–1.28)	4 584	71	1.08 (0.86–1.37)
7–9	1 735	14		1.18 (0.70–2.00)	1 010	32	0.94 (0.67–1.33)	2 745	46	1.02 (0.76–1.36)
10+	891	12		1.20 (0.68–2.11)	498	17	0.64 (0.40–1.04)	1 389	29	0.81 (0.56–1.17)

## Discussion

In our population-based cohort study of mobile phone subscription holders, we found no overall increased risk of MS among subscription holders, irrespective of whether analyzed until clinical MS diagnosis or until first recorded symptom. Neither did we find any overall increased risk of death among subscription holding MS-patients. Among subgroups of women, we did observe some risk increases of MS, MS-symptoms and death after long-term subscription, however, the numbers were small in these analyses and may have been chance findings. Among men, we found a tendency towards increased risks of MS-symptoms, in the first year after mobile phone subscription, and of MS in the period 1–9 years after first subscription. Among male MS patients, the risk of death appeared to be decreased in the first years of mobile phone exposure, approaching unity after 4 years. The first symptoms of MS were different among mobile phone users compared to non-subscribers, with subscription holders having more frequent fatigue among women, increased optic neuritis among men, and diplopia in both sexes.

### Risk of MS-diagnosis in Subscription Holders

Our observation of an increased risk of MS among men 1–9 years after first subscription could be due to reverse causation, particularly since men were somewhat more likely to obtain a subscription in the years immediately after being recorded with their first symptoms. However, as a large proportion of MS cases were diagnosed within the first few years after their first symptom, we would have expected to also see a risk increase in the first year after first subscription. We did observe slightly increased risk of first symptoms of MS within the first year after first subscription. This could be a triggering/promoting effect, but such effects would likely also affect women, contrary to our findings, and it therefore seems more likely a chance finding or detection bias related to use of the mobile phone. In addition, there is also the possibility of reverse causation, since only the year of first recorded symptom and not the actual date of first symptom experienced was available for most subjects, therefore the temporal resolution does not allow exact sequencing of events within the year of first symptom. This interpretation is supported by the fact that we observed both an increased chance of obtaining a subscription in the year of the first symptom and an increased risk of having a first symptom of future MS within the first year after first subscription. Among female mobile phone users, we observed a doubling in risk of MS 10 or more years after first subscription, however, this finding was based on very few cases, and the limited statistical precision in combination with lack of dose response and disagreement with the results in men suggests chance as a more likely explanation. The fact that the risk was also present when analysing until first symptom and that the risk was even more pronounced when the follow-up period was restricted to 13+ years may, however, merit further attention. It should also be kept in mind that the first female users who were the most likely long term exposed subjects were a select subgroup of presumably affluent women, and therefore confounding may also have influenced the results.

### Debut Symptoms

Fatigue was more frequent among female mobile phone subscribers, but not among men. Although tiredness/fatigue is among the more common complaints in mobile users [25], [26], and there is some evidence that RF-EMF from mobile phones influences EEG [27], the lack of consistency in results between genders and the small numbers do not allow firm conclusions based on our data. We observed an increased risk of optic neuritis among male subscribers and diplopia (double vision) was elevated in both genders. A recent laboratory study found no effect on visually evoked potentials in GSM exposed men [28], but effects of short term exposure in a laboratory may differ from effects due to long term exposure. Several small questionnaire-based cross-sectional studies have reported an increased occurrence of blurring of vision (among the symptoms of both optic neuritis and diplopia) among mobile phone users, although the impact of participation and reporting biases should be considered in the interpretation of these studies [29]–[32]. Also, self-reported “blurring of vision" is a very vague endpoint, compared to the MS-registry which contains, thoroughly validated, records of symptoms persisting for longer periods. Although the overall composition of symptoms in our study did not differ significantly by exposure status for men, the uniformly increased risk in both genders for diplopia indicates that these observations merit further study. If future studies corroborate that the symptom picture differs, it should be considered that the visual nerve may be in the exposure field, but also that the battery in mobile phones heats up when the phone is in operation and that heating may elicit symptoms in MS-patients [33], [34].

### Risk of Death in MS-patients

We did not observe an increased risk of death in MS cases following mobile subscription, rather, among men, there was a tendency for a decreased risk of death in the first years after obtaining a subscription. These estimates were, however, based on small numbers, and the risk decrease among men is likely attributable to chance. It is, however, conceivable that there is a healthy subscriber bias at the time of mobile phone acquisition as its use requires the physical and cognitive capabilities to operate the mobile phone as well as spending enough time away from a landline phone to justify the extra expense. The numbers of deaths among female MS patients with mobile phone subscription were too small to allow any conclusions.

### Likelihood of Subscribing for a Mobile Phone in MS Patients

It is a general epidemiological concern that early symptoms of the outcome of interest may have a reverse effect on the likelihood of exposure and thereby make it difficult to discern cause and effect. For instance, migraine-like symptoms are more common in MS-patients [35], and the Danish Migraine Association has been advising migraine patients to obtain a mobile phone to facilitate access to help in case of an attack. For women in our study, the likelihood of obtaining a subscription was only increased more than one year after being diagnosed with MS, indicating that for women the disease may indeed influence the likelihood of getting a subscription but only during a period of little impact on the risk analyses. Among men, there was no effect of being diagnosed with MS on the likelihood of obtaining a subscription; there was, however, some indication of an increased likelihood of getting a subscription in the first four years after being diagnosed with the first symptoms of MS. This means that, among men, reverse causation bias is a potential problem in our analyses, especially since the effect may be present even before the year of the first recorded symptoms, if prodromal symptoms exist. Data on this issue were, however, sparse and the effect estimates did not reach significance. It is therefore not possible to substantiate if the observed associations are chance findings or true associations applicable to other studies.

### Strengths and Limitations

Our study was a nationwide cohort study based on objective and prospectively registered exposure data with validated and carefully evaluated outcome data from a nationwide high quality register [18]. The applied register approach practically eliminates loss to follow-up and provides accurate and virtually complete nationwide ascertainment of MS-cases. The validated diagnoses in the MS-register allowed more reliable estimates than in the previous publication of the same exposure cohort where MS was assessed from hospital admissions [15]. In addition, the details on type and date of early symptoms of MS from the MS-registry also allowed us to explore if mobile usage had any impact on the symptom pattern or course of the disease, although we only had the year of first symptom as recorded retrospectively from medical journals and not the actual date of the first symptoms experienced. Furthermore, the exposure assessment was improved in our study by left-truncating exposure data to 1987, thereby reducing misclassification of exposure due to car phones that were available in Denmark since 1982, but in terms of exposure to the head are several orders of magnitude lower than handheld phones.

A limitation of the study was the lack of exposure details and potential exposure misclassification [36]. Subscription holders not using their mobile phone may erroneously be classified as exposed or vice versa. However, according to the annual report of the Danish national IT and Telecom Agency, the average annual outgoing traffic per subscription was around 1400 min/subscription until 1992 when it started to decrease, stabilizing at around 900 min/subscription from 1995 to 2002 [37]. This change presumably reflects that the very early users had sufficient need of the mobile phone to justify the expensive subscription. When examining risk at 10+ or even 13+ years of follow-up, where the exposure contrast was the largest, we found no increased risks of MS, MS-symptoms or death among men. Among women, however, we did see indications of increased risks for the various outcomes among long-term subscribers, although the numbers were too small to allow reliable interpretation. For several outcomes, we observed somewhat different associations among men and women. Although we cannot rule out gender differences, differences in residual confounding seem more likely given the fact that the uptake of mobile phones was so different, in time and potentially in reasons, combined with the established gender difference in MS incidence [38].

### Conclusions

We found little evidence for an association between mobile phone use and risk of MS or of death in MS patients in this nationwide study of mobile phone subscribers in Denmark. We did, however, observe an increased risk of both MS symptoms and diagnosis among long-term female subscribers, although this was based on small numbers. The presenting symptoms of MS differed between subscribers and nonsubscribers with elevated risks for symptoms previously suggested to be associated with mobile phone use (visual disturbances and fatigue) observed among the mobile phone users. These observations merit further attention in more detailed studies, such as the ongoing prospective COSMOS study [39], which will allow more detailed exposure assessment, collecting information on potential confounders and detailed chronological information to identify potential reverse causation bias.
